# Progressive mitochondrial dysfunction impairs visual signal transduction and induces retinal degeneration in *Drosophila*

**DOI:** 10.3389/finsc.2025.1596020

**Published:** 2025-07-28

**Authors:** Jinguo Cao, Yining Li, Xiaohui Hu, Zhaoqi Wu, Jiting Zhang, Yue Zhou, Wei Luan, Wen Hu, Jianhong Tang

**Affiliations:** ^1^ Key Laboratory of Mitochondrial Medicine, Key Laboratory of Genetic and Developmental Related Diseases, Department of Basic Medicine, Gannan Medical University, Ganzhou, China; ^2^ Department of Basic Medicine, Gannan Health Vocational College, Ganzhou, China; ^3^ Cambridge-Suda Genomic Resource Center, Suzhou Medical College, Soochow University, Suzhou, China; ^4^ Key Laboratory of Prevention and Treatment of Cardiovascular and Cerebrovascular Diseases, Ministry of Education, Scientific Research Center, Gannan Medical University, Ganzhou, China

**Keywords:** mitochondrial dysfunction, retinitis pigmentosa, *Drosophila*, phototransduction, reactive oxygen species

## Abstract

Mitochondrial dysfunction is closely associated with the pathogenesis of retinitis pigmentosa (RP), often through the generation of reactive oxygen species (ROS), which disrupts visual signal transduction. However, in certain instances, mitochondrial dysfunction does not correlate with an increase in ROS, and the precise mechanisms by which mitochondrial dysfunction contributes to RP remain poorly understood. In this study, we demonstrate that mitochondrial dysfunction can also impair visual signal transduction through ROS-independent mechanisms. Specifically, we identify that mitochondrial dysfunction affects key processes in phototransduction, including activation and bleaching, leading to the degradation of photoreceptor proteins and, ultimately, retinal degeneration. Our findings reveal that mitochondrial dysfunction influences RP through multifaceted pathways, underscoring its role in both hereditary and age-related forms of visual diseases. This study enhances our understanding of the molecular mechanisms underlying RP and establishes a novel model for investigating mitochondrial dysfunction in visual pathologies.

## Introduction

Retinitis pigmentosa (RP) is a hereditary retinal disorder characterized by the progressive degeneration of photoreceptor cells, which ultimately leads to debilitating visual impairments such as night blindness, tunnel vision, and eventual vision loss (1, 2). RP exhibits considerable genetic heterogeneity, manifesting in various inheritance patterns, including autosomal dominant (15%-25%), autosomal recessive (5%-20%), X-linked forms (10%-15%) (3–6). Additionally, the remaining 40–50% of cases display heterogeneous clinical and genetic features, including rare forms such as digenic RP and maternally inherited mitochondrial RP. To date, more than 200 genes have been associated with RP, many of which are involved in fundamental biological processes such as phototransduction, mitochondrial function, metabolism, and cellular stress response (7–9).

Among the multiple factors contributing to RP, mitochondrial dysfunction has emerged as a critical player in disease progression (10–12). Mitochondrial-related optic neuropathies, including Leber’s hereditary optic neuropathy and autosomal dominant optic atrophy, further underscore the central role of mitochondria in retinal health (13–15). Recent study demonstrated that the accumulation of mtDNA mutations in PolG mutant mice results in impaired retinal function, heightened sensitivity to stress, and accelerated neurodegeneration. These findings underscore a potential connection between mitochondrial dysfunction and age-related neuronal deterioration (16). Notably, mitochondrial dysfunction in RP is frequently associated with an increase in reactive oxygen species (ROS), which have been identified as major contributors to retinal cell damage and degeneration (17–21). Beyond the ROS-induced mechanisms, however, other aspects of mitochondrial dysfunction, particularly the gradual accumulation of defective mitochondria over time, remain underexplored in the pathogenesis of RP. Meanwhile despite the well-established role of nuclear gene mutations in causing mitochondrial dysfunction in RP, the influence of progressive mitochondrial dysfunction on visual signal transduction remains poorly understood and warrants further investigation. This gap in knowledge is particularly significant because, in addition to these nuclear mutations, the gradual accumulation of dysfunctional mitochondria over time could potentially exacerbate retinal degeneration. To address this, model organisms such as *Drosophila melanogaster* have proven invaluable. These organisms harbor many genes that are conserved in humans and have been instrumental in elucidating the underlying mechanisms of RP (22–24). The *Drosophila* compound eye consists of approximately 800 hexagonally arranged units known as ommatidia, each comprising 20 cells, including eight photoreceptor neurons. Each photoreceptor cell contains a specialized microvillar structure called the rhabdomere, which serves as the functional equivalent of the outer segments of vertebrate rods and cones (25, 26). In *Drosophila*, visual transduction begins with the photon-induced isomerization of the chromophore within rhodopsin, which in turn activates a heterotrimeric G protein. This activation triggers the effector enzyme phospholipase Cβ (PLC) (27), ultimately leading to the opening of the TRP and TRPL cation channels.

In this study, we utilized a *Drosophila melanogaster* model with the *mt:CoI^ts^* mutation to investigate how progressive mitochondrial dysfunction impacts visual signal transduction in RP (28). The *mt:CoI^ts^* mutation affects a subunit of mitochondrial complex IV (Mitochondrial respiratory chain complex IV, cytochrome c oxidase), with mitochondrial function remaining intact at 18°C. However, when flies are raised at 29°C, they fail to hatch, and adult flies die within five days (29). Our experiments show that exposure to 29°C leads to a gradual decline in mitochondrial activity in newly emerged flies, which is reflected by a reduction in the amplitude of light-evoked responses, impaired light adaptation, and decreased sensitivity to light. Electron microscopy revealed that mitochondrial dysfunction resulted in light-dependent retinal degeneration, primarily due to the formation of stable Rh1-Arr2 (Rhodopsin-Arrestin2) complexes. These findings suggest that, in addition to nuclear gene mutations, the gradual loss of mitochondrial function can disrupt visual signal transduction and contribute to RP. Moreover, our results indicate that different mitochondrial diseases may follow distinct progression pathways, leading to varying effects on visual function.

## Results

### *mt: CoI^ts^* flies show normal electroretinogram responses at 18°C

To investigate the role of mitochondria in phototransduction, we utilized a mitochondrial mutant (*mt: CoI^ts^*) to examine the effects of mitochondrial dysfunction on visual transduction. This mutant line contains a temperature sensitive point mutation in the mitochondrial gene *mt: CoI*.

**Figure 1:**
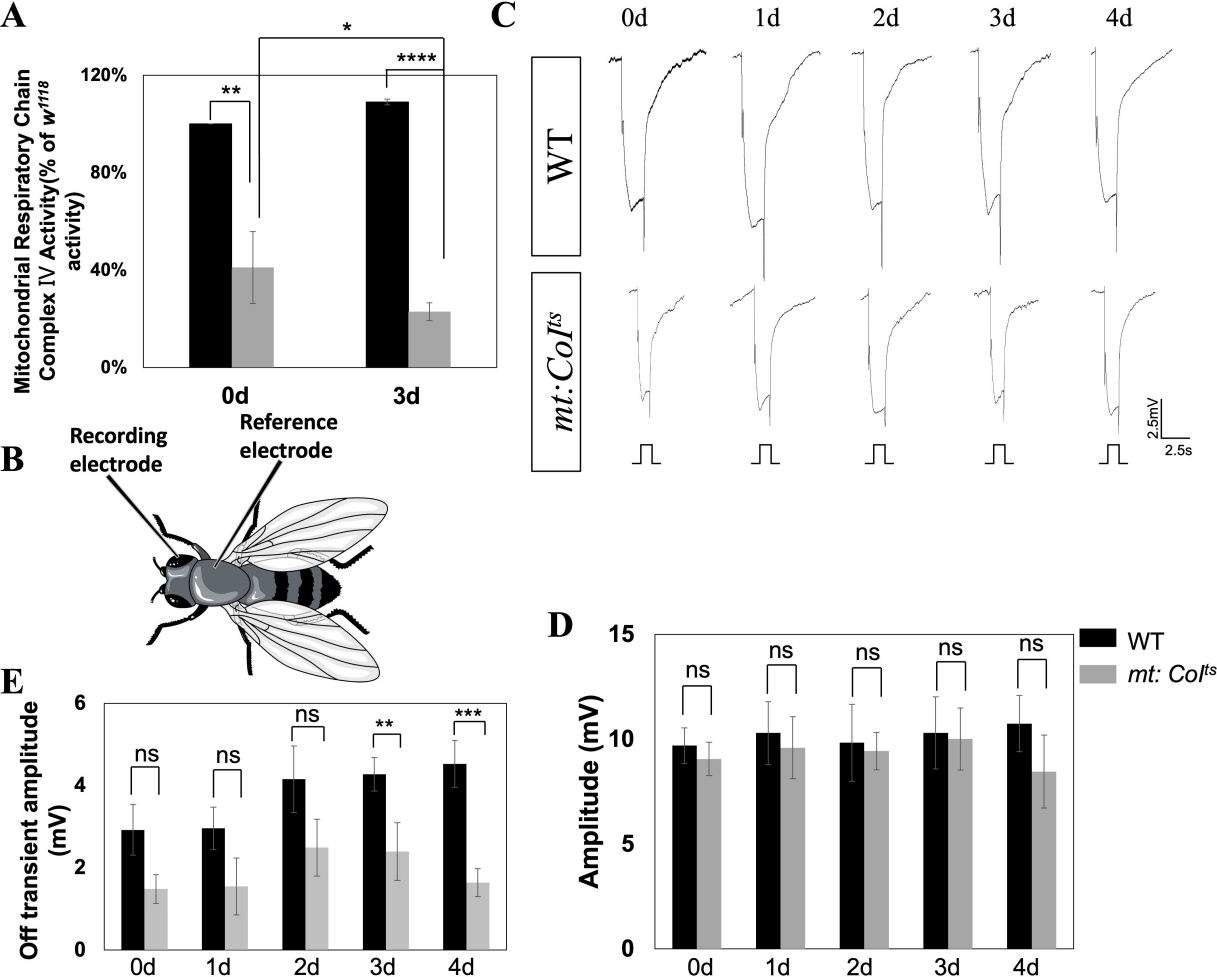
Impaired mitochondrial function can lead to phototransduction deficits in *mt:CoI^ts^* mutant flies. **(A)** Quantitative comparison of mitochondrial respiratory chain complex IV activity in wild-type and *mt:CoI^ts^* mutant flies at days 0 and 3 post-eclosion at 29 °C. *mt:CoI^ts^* mutants exhibit a significant reduction in complex IV activity compared to WT at both day 0 (** *P* < 0.01) and day 3 (**** *P* < 0.001). Data are expressed as percentage activity relative to WT and presented as mean ± SEM. Statistical significance was assessed using unpaired *t*-tests. Three independent biological replicates were analyzed, each consisting of 40 heads per genotype. **(B)** Schematic illustration of the ERG recording. A glass microelectrode is placed on the surface of the compound eye to record the light-evoked potentials. A reference electrode is placed on the thorax to complete the electrical circuit. **(C)** Representative electroretinogram (ERG) traces recorded from WT and *mt:CoI^ts^* mutants aged 0–4 days under 2.5 s light stimulation. WT flies maintain robust photoreceptor responses, and *mt:CoI^ts^* mutants exhibit normal ERG responses like WT flies. Scale bar: 2.5 mV, 2.5 s. **(D)** Quantitative analysis of ERG amplitudes from day 0 to day 4 post-eclosion. No significant differences in ERG amplitude were detected between WT and *mt:CoI^ts^* mutants at any time point examined. Data are presented as mean ± SEM. Statistical comparisons were performed using unpaired *t*-tests, ns denotes no statistically significant difference. A minimum of five flies were analyzed per group. **(E)** Quantification of ERG off-transient amplitudes in WT and *mt:CoI^ts^* mutants from day 0 to day 4 post-eclosion. *mt:CoI^ts^* mutants exhibit significantly reduced off-transient amplitudes at day 3 (** *P* < 0.01) and day 4 (*** *P* < 0.005) compared to WT. Statistical analyses were performed using unpaired *t*-tests. ns indicates no statistically significant difference. A minimum of five flies were analyzed per group.

In the *mt: CoI^ts^* mutant, CoI protein remains normal at a low temperature (18°C) but becomes unstable at a restrictive temperature (29°C), leading to a reduction in complex IV activity. As shown in **Figure 1A**, in *mt: CoI^ts^* mutant flies reared at 29°C, complex IV activity is significantly reduced compared to wild-type, with activity decreasing to ~41% of wild-type levels at day 0 and ~23% at day 3. At 18°C, the CoI protein in the *mt: CoI^ts^* mutant functions normally, and mitochondrial complex IV remains unaffected.

The most sensitive exam for phototransduction is the ERG recording, a simple electrophysiological test, shown in **Figure 1B**. Electroretinograms (ERGs) are extracellular recordings that capture the aggregate electrical responses of retinal cells to light stimuli. They are commonly conducted using white light, with a recording electrode positioned on the surface of the compound eye (26). Under this condition, the ERG responses of flies are comparable to wild-type flies, as shown in **Figure 1C**. We recorded ERG responses in wild-type and mutant flies reared at 18°C from eclosion (day 0) to day 4. **Figures 1D, E** showed the quantified ERG amplitudes and the size of the off-transient. ERG amplitudes reflect the overall activity of retinal cells, while the off-transient component arises from postsynaptic responses in the lamina, downstream of photoreceptor signaling. While ERG amplitudes showed no significant differences, the off-transient was slightly reduced at days 3–4. ERG responses reflect the photoreceptor cells’ reaction to light stimuli, with normal ERG responses indicating an intact phototransduction pathway within the photoreceptor cells in the *mt: CoI^ts^* mutants.

### *mt: CoI^ts^* flies show normal light sensitivity and Rhodopsin level at 18°C

**Figure 2:**
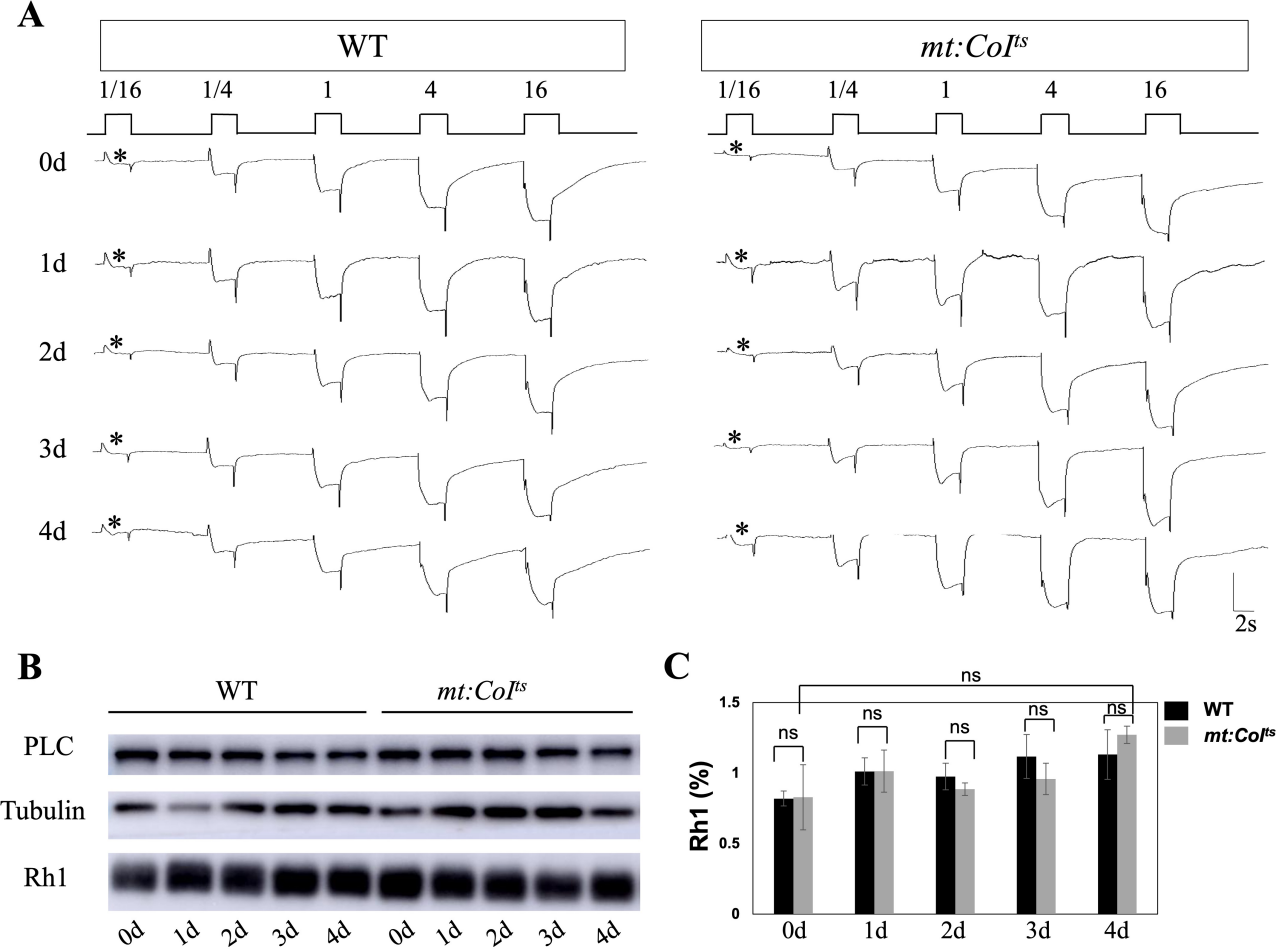
Phototransduction responses and Rh1 protein levels are maintained in *mt:CoI^ts^* mutants. **(A)** Representative ERG recordings in wild-type and *mt:CoI^ts^* mutant flies aged 0–4 days post-eclosion at 18°C. Flies were exposed to increasing light intensities (1/16, 1/4, 1, 4, and 16). The first light response in each condition is marked with an asterisk (*). *mt:CoI^ts^* flies exhibit robust and consistent light responses across all light intensities as wild-type flies. Scale bar: 2 s. **(B)** Western blot analysis of Rh1 and PLC protein levels in WT and *mt:CoI^ts^* mutants at days 0–4 post-eclosion. Tubulin serves as internal loading controls. Rh1 and PLC protein levels remain relatively stable across all time points in both WT and *mt:CoI^ts^* mutants. **(C)** Quantification of Rh1 protein levels in WT and *mt:CoI^ts^* mutants from day 0 to day 4 post-eclosion. Rh1 levels were normalized to Tubulin. No statistically significant differences were detected between genotypes at any time point examined (unpaired *t*-tests; ns indicates no significant difference). Data are presented as mean ± SEM. Three independent biological replicates were analyzed, each comprising 10 heads per genotype.

Light sensitivity reflects the capacity to detect low-intensity light and provides an alternative approach for evaluating visual function. Light sensitivity tests were performed in dark-reared 0–4 day wild-type and *mt: CoI^ts^* mutants and no reduction in light sensitivity were observed in *mt: CoI^ts^* mutants, as shown in **Figure 2A**. The results showed that the *mt: CoI^ts^* mutants retained normal sensitivity to light under low-light conditions at 18°C. In photoreceptor cells, membrane-bound rhodopsin receives light stimuli and activates Gq, which in turn activates downstream PLC (phospholipase Cβ), leading to the opening of TRP/TRPL ion channels (26). Therefore, we measured the levels of key components involved in phototransduction, rhodopsin (Rh1) and PLC, in wild-type and *mt: CoI^ts^* mutant flies. As shown in **Figures 2B, C**, the levels of Rh1 and PLC remained unchanged in the mutants.

In summary, at 18°C, where mitochondrial function is uncompromised, the ERG response, light sensitivity, and the abundance of key phototransduction molecules in the *mt: CoI^ts^* mutants are indistinguishable from those in wild-type flies.

### *mt: CoI^ts^* flies show defect ERG responses at 29°C

**Figure 3:**
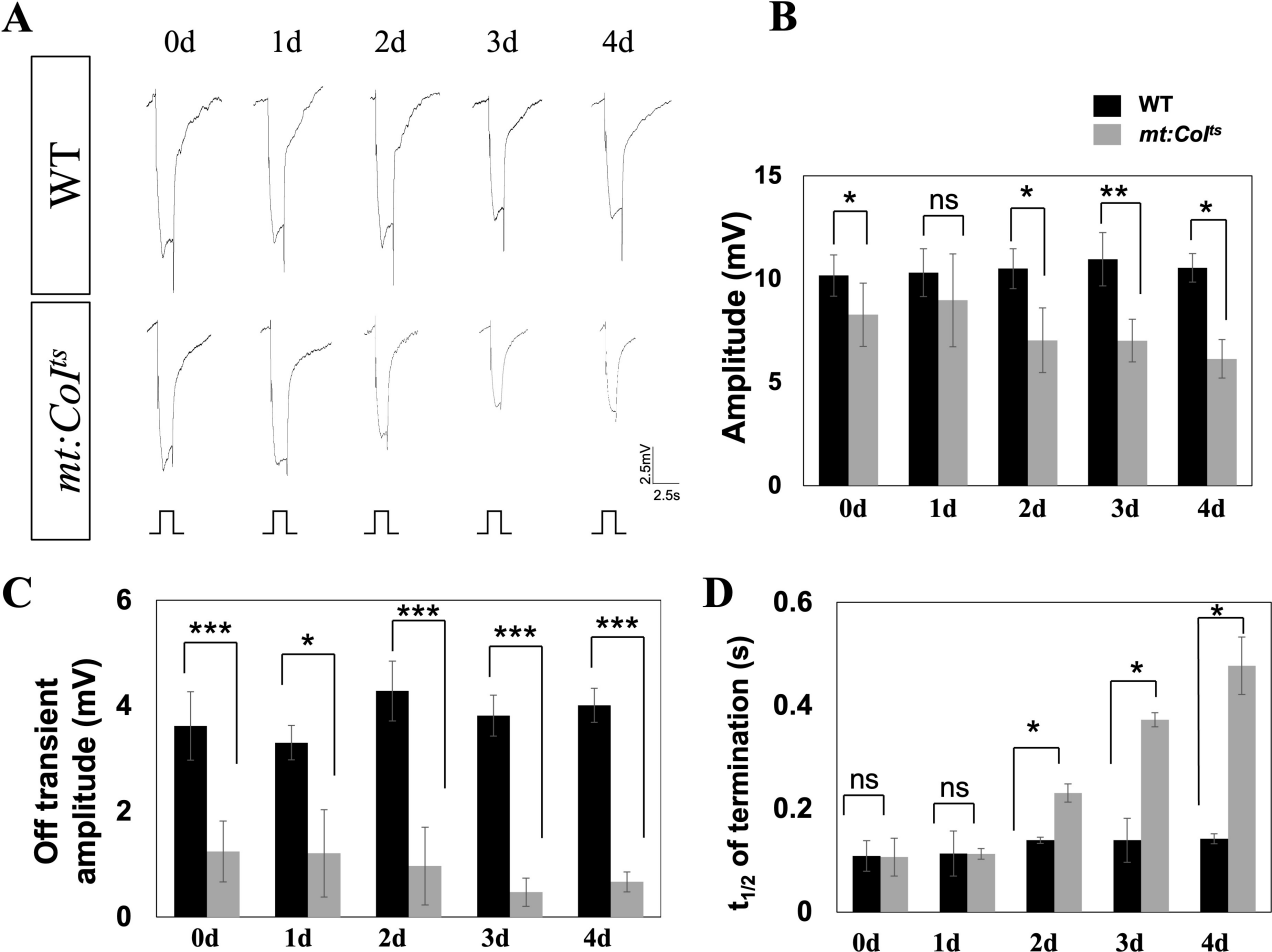
Electrophysiological analysis reveals progressive deficits in *mt:CoI^ts^* mutants over time. **(A)** Representative electroretinogram (ERG) traces from wild-type and *mt:CoI^ts^* mutant flies aged 0–4 days post-eclosion at 29°C. Flies were subjected to a 2.5 s light stimulus. Wild-type flies exhibit stable light responses, while *mt:CoI^ts^* mutants show a progressive reduction in response amplitude over time. Scale bar: 2.5 mV, 2.5 s. **(B)** Quantification of ERG response amplitudes in wild-type and *mt:CoI^ts^* mutants at 0–4 days post-eclosion. The amplitude remains relatively stable in wild-type flies, whereas a gradual decline is observed in *mt:CoI^ts^* mutants with increasing age. Data are expressed as mean ± SEM. Statistical comparisons were performed using unpaired *t*-tests, * *P* < 0.05, * * *P* < 0.01, ns denotes no statistically significant difference. A minimum of five flies were analyzed per group. **(C)** Quantification of ERG off-transient amplitudes, reflecting synaptic transmission between photoreceptors and downstream neurons, in wild-type and *mt:CoI^ts^* mutants at 0–4 days post-eclosion. *mt:CoI^ts^* mutants exhibit significantly reduced off-transient amplitudes. Data are expressed as mean ± SEM. Statistical comparisons were performed using unpaired *t*-tests, * *P* < 0.05, *** *P* < 0.005. A minimum of five flies were analyzed per group. **(D)** Quantification of the half-recovery time following light stimulation in wild-type and *mt:CoI^ts^* mutants at 0–4 days post-eclosion. While wild-type flies maintain stable recovery kinetics, *mt:CoI^ts^* mutants show a progressive delay in recovery time, suggesting deficits in phototransduction shutoff mechanisms. Data are expressed as mean ± SEM. Statistical comparisons were performed using unpaired *t*-tests, * *P* < 0.05, ns denotes no statistically significant difference. A minimum of five flies were analyzed per group.

Previous studies have reported that mutations in various genes regulating vision can lead to changes in the electroretinogram (ERG), with some phenotypes being severe, while others are more moderate (26). Our focus in the following investigation is to determine whether mitochondrial dysfunction affects the ERG response. To induce mitochondrial dysfunction in the *mt: CoI^ts^* mutants, we reared the flies at 29°C and performed ERG recordings from 0 to 4 days post-eclosion. We observed a significant difference in the ERG response between *mt: CoI^ts^* mutants and wild-type flies, as shown in **Figure 3A**, with the disparity becoming more pronounced over days. Statistical analysis revealed that the ERG amplitude in *mt:CoI^ts^* mutants progressively decreased with age, showing an approximately 40% reduction by day 4, as depicted in **Figure 3B**. Additionally, *mt:CoI^ts^* mutants exhibited a reduced off-transient and a delayed photoresponse termination phenotype (**Figures 3C, D**). The off-transient which results from postsynaptic activity in the lamina, downstream of photoreceptor cell signaling was significantly diminished, with a reduction of approximately 83% by day 4, and the photo-response termination time was approximately five times longer than in wild-type flies. t_1_/_2_ reflects the termination speed of the photoresponse. Our findings reveal that mitochondrial dysfunction is closely associated with both the light activation and deactivation processes.

Mitochondrial dysfunction is often associated with elevated levels of reactive oxygen species (ROS) in retinitis pigmentosa (RP). However, *Zhe Chen* et al. reported that ROS levels in *mt:CoI^ts^* flies were comparable to those in wild-type flies (30). These findings suggest that the phototransduction defect observed in *mt:CoI^ts^* flies is not attributable to increased ROS production.

### *mt: CoI^ts^* flies show reduced light sensitivity and rhodopsin level at 29°C

**Figure 4:**
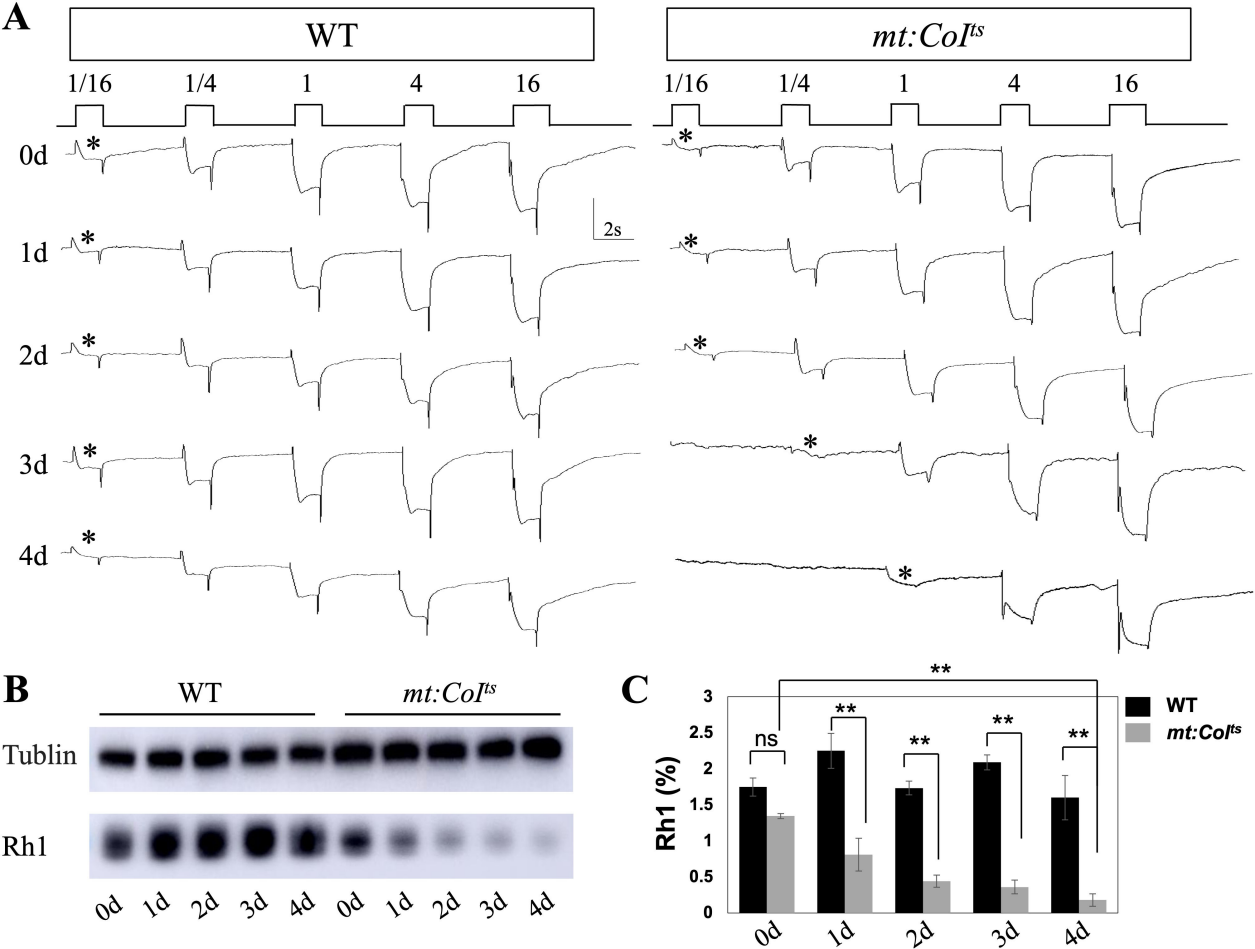
Progressive impairment of photoreceptor function and Rh1 levels in *mt: CoI^ts^* mutants over time. **(A)** Representative electroretinogram (ERG) recordings from wild-type and *mt: CoI^ts^* mutant flies aged 0–4 days post-eclosion at 29°C under increasing light intensities. The initial light response is marked with an asterisk (*). Wild-type flies exhibit stable and robust light responses across all light conditions, while *mt: CoI^ts^* mutants show a progressive reduction in light response sensitivity. **(B)** Western blot analysis of Rh1 protein levels in wild-type and *mt: CoI^ts^* mutant flies aged 0–4 days post-eclosion. Tubulin serve as loading controls. A notable reduction in Rh1 levels is observed in *mt: CoI^ts^* mutants compared to wild-type controls. **(C)** Quantification of Rh1 protein levels in wild-type and *mt: CoI^ts^* mutant flies aged 0–4 days post-eclosion. Rh1 protein levels were normalized to loading controls and expressed as grayscale intensity. Data are presented as mean ± SEM. Statistical significance was determined using unpaired *t* tests, * P < 0.05, ** P < 0.01, ns indicates no statistical significance. Three independent biological replicates were analyzed, each comprising 10 heads per genotype.

Previous studies have indicated that ERG abnormalities are closely related to changes in the levels of the photoreceptor Rh1 (31, 32), and alterations in Rh1 content can lead to variations in light sensitivity. In the following experiments, we aim to investigate whether mitochondrial dysfunction affects light sensitivity and Rh1 levels. Light sensitivity assays conducted on wild-type and *mt:CoI^ts^* mutants reared at 29°C revealed that the *mt:CoI^ts^* flies displayed reduced light sensitivity, with this diminished phenotype becoming more pronounced with age (**Figure 4A**). When we measured rhodopsin levels using Western blot, we found a significant decrease in its abundance. By 1-day post-eclosion, the rhodopsin content had already dropped to approximately 36% of the wild-type level, and by day 4, it was about 11% of the wild-type level (**Figures 4B, C**). These results suggest that mitochondrial dysfunction leads to severe defects in phototransduction, indicating that the proper maintenance of the phototransduction pathway requires healthy mitochondria.

### *mt: CoI^ts^* flies undergo retinal degeneration

**Figure 5:**
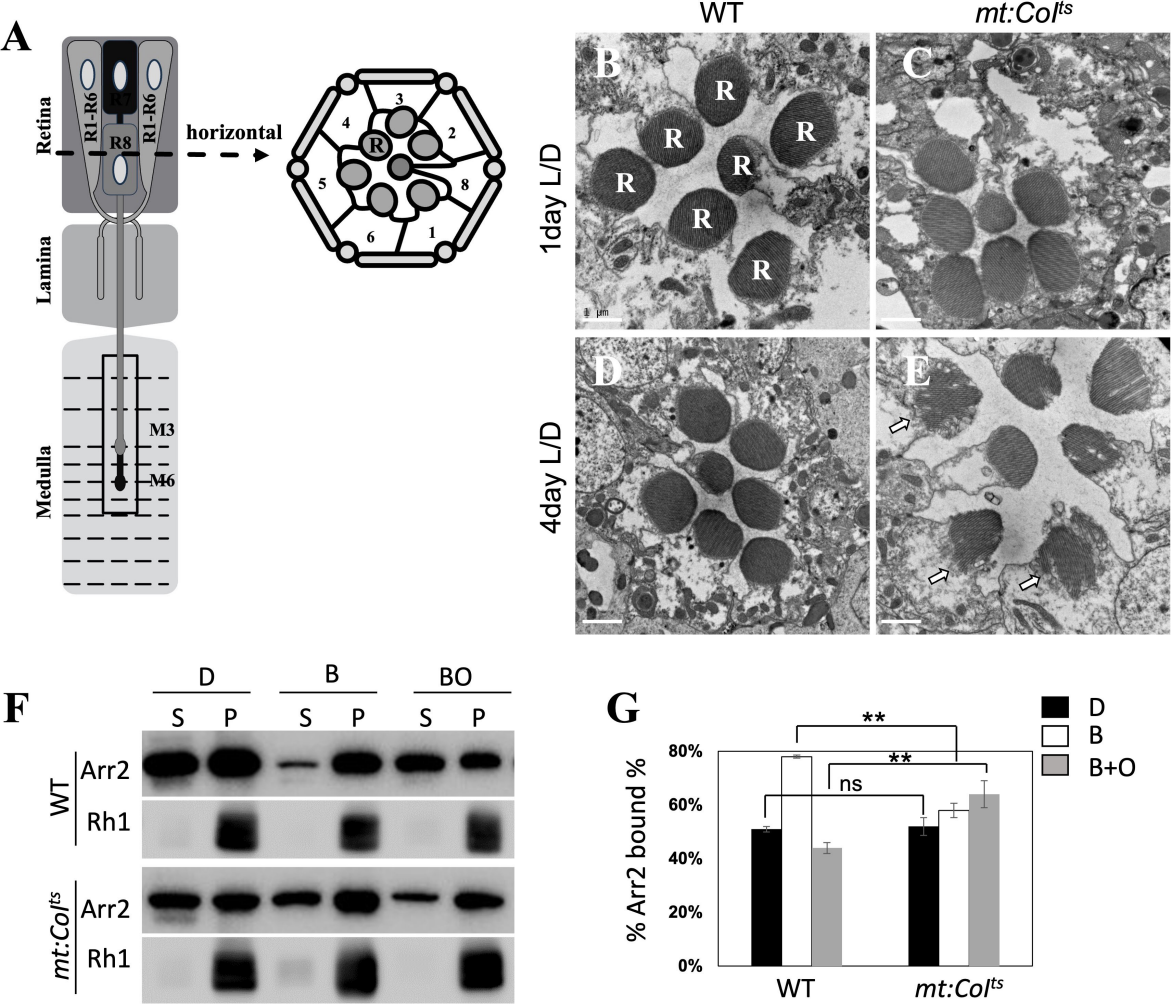
*mt:CoI^ts^* mutant flies undergo retinal degeneration. **(A)** The left schematic shows a single ommatidium. Each ommatidium comprises eight photoreceptor neurons (R1–R8). Axons from R1–R6 project as a bundle to the lamina, while R7 and R8 extend axons to distinct layers within the medulla. R1–R6: outer photoreceptors; R7 and R8: inner photoreceptors targeting the medulla. Right: Horizontal cross-section of an ommatidium, showing the spatial arrangement of the eight photoreceptor cell bodies (R1–R8). R: Rhabdomere—an array of microvilli enriched in rhodopsin that mediates photon detection and initiates visual signal transduction. **(B, C)** Electron microscopy (EM) images of retinal cross-sections from wild-type **(A)** and *mt: CoI^ts^* mutant flies **(B)**. At 1 day post-eclosion, wild-type and *mt:CoI^ts^* mutant flies maintain a well-organized and compact rhabdomere structure. Scale bar: 1 μm. **(D, E)** EM images of the wild-type **(C)** and *mt:CoI^ts^* mutant **(D)** retinas confirm the progressive retinal degeneration in *mt: CoI^ts^* mutants at 4 day post-eclosion. Scale bar: 1 μm. The white arrow points to the rhabdomeres with a loosened or fragmented structure. **(F)** Western blot analysis of Arr2 translocation in wild-type and *mt:CoI^ts^* mutant flies under three conditions: darkness **(D)**, after blue light exposure **(B)**, and subsequent orange light treatment (BO). Protein levels were assessed to evaluate light-induced Arr2-Rh1 complex formation and dissociation. **(G)** Quantification of the percentage of Arr2 bound to Rh1 under the same conditions (darkness, blue light, and orange light) in wild-type and *mt:CoI^ts^* mutant flies. Under dark conditions, the percentage of Arr2 bound to Rh1 in *mt: CoI^ts^* mutant flies was comparable to that in wild-type flies. However, after blue light exposure, this percentage was significantly reduced in *mt: CoI^ts^* mutants compared to wild type, but markedly increased when orange light was applied following the blue light treatment. Data are presented as mean ± SEM. Statistical significance was determined using two-way ANOVA, ** *P* < 0.01, ns indicates no statistical significance. Three independent biological replicates were analyzed, each comprising 10 heads per genotype.

In *Drosophila*, mutations affect phototransduction cascades can lead to light-dependent retinal degeneration (26, 33, 34). Therefore, we aimed to investigate whether the impaired light signal transduction resulting from mitochondrial dysfunction could contribute to retinal degeneration. We used electron microscopy to analyze the ultrastructure of photoreceptor cells. The schematic shows a single ommatidium (**Figure 5A**). Each ommatidium comprises eight photoreceptor cells (R1–R8). Horizontal cross-section of an ommatidium, showing the spatial arrangement of the eight photoreceptor cell bodies (R1–R8). Rhabdomere is an array of microvilli enriched in rhodopsin that mediates photon detection and initiates visual signal transduction. In transmission electron micrographs of wild-type flies, rhabdomeres are observed as well-defined, electron-dense circular profiles, denoted by the label R (**Figure 5B**). We found that, at 4 days post-eclosion, mutants reared at 29°C exhibited retinal degeneration (**Figure 5E**), a phenotype not observed in 1 or 4 day-old wild-type flies (**Figures 5B, D**). The white arrows in **Figure 5E** highlight disrupted rhabdomere architecture, indicative of ongoing retinal degeneration. Additionally, 1 day-old mutants reared at 18°C did not show similar degeneration (**Figure 5C**), confirming that the retinal degeneration is indeed caused by accumulated mitochondrial dysfunction.

Light stimulation triggers Ca²^+^ influx, which activates CaM kinase II, leading to the phosphorylation of Arr2 and subsequent release of Arr2 from Rh1 (35, 36). In *mt:CoI^ts^* mutants, the impaired Ca²^+^ influx (30) may slow down the release of Arr2 from Rh1, resulting in the accumulation of stable Arr2-Rh1 complexes, ultimately contributing to retinal degeneration. Hence, we investigated whether the same mechanism contributes to retinal degeneration in *mt:CoI^ts^* mutants. Exposure to 480 nm blue light converts Rh1 to metarhodopsin, promoting its binding with Arr2. Metarhodopsin can then be photoconverted back to inactive rhodopsin with 550 nm orange light, leading to the release of Arr2 (37). In wild-type flies, exposure to blue light results in 78% binding of Arr2 to Rh1, with 56% of Arr2 released from Rh1 following orange light exposure (**Figures 5F, G**). In contrast, in the *mt:CoI^ts^* mutant, blue light exposure induces only around 58% binding between Arr2 and Rh1, and 36% release of Arr2 from Rh1 after orange light treatment (**Figures 5F, G**). Our results indicate that mitochondrial dysfunction leads to the formation of a stable Arr2-Rh1 complex in *mt:CoI^ts^* mutants, ultimately resulting in retinal degeneration.

## Materials and methods

### Fly stocks

The fly strains used in this study are wild-type (*w^1118^*), *mt: CoI^ts^* (gifted by Dr. Xu Hong). Flies were raised in an approximate 12 h light (700 lux)/12 h dark cycle, at temperature of 18°C. After eclosion at 18°C, wild-type and *mt: CoI^ts^* mutants were immediately transferred to either 18°C or 29°C for culturing, with the day of transfer designated as day 0.

### Electroretinogram recording

Electroretinogram (ERG) recordings were conducted following previously established methods (38). In brief, the eyes of the flies were subjected to 5-second light pulses (4000 lux). For each genotype and experimental condition, more than 10 flies were tested. To assess the response termination speed, the time required to reach half-recovery was recorded, and the standard error (S.E.) was calculated. t½ is defined as the time taken for the response to recover by 50% after the cessation of light stimulation.

The measurement of relative light sensitivity was performed according to the protocol described in (39). For each genotype and experimental condition, 10 flies were analyzed.

### Western blotting

Fly heads were lysed in SDS-sample buffer, and the proteins were separated by SDS-PAGE before being transferred onto PVDF membranes in Tris-glycine buffer. The membranes were incubated with primary antibodies, including mouse anti-Rh1 (1:3000 dilution, DSHB, 4C5), rabbit anti-Arr2 (1:1000 dilution, C. Montell), mouse anti-Tubulin (1:2000, DSHB, E7) and rabbit anti-PLC (1:1000 dilution, C. Zuker). Following primary antibody incubation, the membranes were probed with either anti-rabbit or anti-mouse IgG horseradish peroxidase conjugates (Abcam ab205718, ab205719). Signal detection was carried out using ECL reagents (Amersham Biosciences, RPN2209).

### Mitochondrial complex IV enzyme activity assay

Approximately 40 wild-type and mutant flies were collected under light/dark conditions (0 days and 3 days post-hatch) and decapitated to isolate the heads. Mitochondrial fractions were prepared using the Qproteome Mitochondria Isolation Kit (Qiagen, 37612). Mitochondrial complex IV activity was assessed using a mitochondrial respiratory chain complex IV assay kit (Sangon Biotech, D799473) with a UV spectrophotometer (Eppendorf Bio Spectrometer). Mitochondrial complex IV activity was calculated based on the absorbance readings and the protein concentration from the bca assay.

### Electron microscopy

EM was conducted following previously established methods (38). Fly heads were fixed in 2.5% glutaraldehyde in 0.1 M sodium cacodylate (pH 7.2) at 4°C for 12 hours. After fixation, the tissue was rinsed three times with 0.1 M sodium cacodylate and then stained with 1% osmium tetroxide for 1 hour at room temperature. A standard dehydration process was carried out using a series of ethanol washes, followed by two 10-minute immersions in propylene oxide. The tissue was then embedded according to standard procedures. For electron microscopy, thin sections (100 nm) were cut, placed onto copper support grids, and stained with uranyl acetate, followed by lead citrate. Micrographs were captured at 80 kV using a Hitachi-7650 microscope.

### Arr2 translocation assay

For each experimental group and condition, eight adult flies were collected and kept in complete darkness with food for over 2 hours to adapt. Following adaptation, the flies were exposed to pure blue light (480 ± 10 nm) for 60 seconds. Fly heads were then isolated using liquid nitrogen and homogenized in the dark. After centrifugation at 14,600 × g for 5 minutes, the pellet and supernatant fractions were separated under dim red light for subsequent Western blot analysis. Arr2 release assays were performed in the same manner, with the exception that the flies were exposed to 60 seconds of pure blue light followed by 2 minutes of pure orange light (580 ± 10 nm).

### Statistics

Quantification of Western blot images was performed using ImageJ software for each quantitative analysis. The data were averaged over three independent trials, and the mean values are presented with standard error (S.E.) as error bars.

## Discussion

In our study, we used *mt:CoI^ts^* mutant fly as a model to investigate the progressive dysfunction of mitochondrial function and its impact on vision. We demonstrate that while mitochondrial function is uncompromised at lower temperatures (18°C), defects in mitochondrial complex IV caused by elevated temperatures (29°C) significantly impair visual function, leading to severe retinal degeneration.

### The *mt:CoI^ts^* mutant fly is an excellent model for studying the mitochondrial function on vision

Mitochondrial dysfunction is a critical factor in retinal degeneration, a condition that has attracted significant attention due to its complex pathophysiology (11, 40). Numerous studies have implicated mutations in mitochondrial function-related genes as key contributors to retinal degeneration. Nevertheless, a key distinction exists: while some forms of mitochondrial dysfunction are induced by genetic mutations, most are due to the accumulation of abnormal mitochondria. This form of dysfunction, exacerbated by the lack of effective repair mechanisms and histone protection, is particularly prone to occurrence, yet its implications for visual signal transduction and the onset of RP remain less well understood.

Zhe Chen et al. used a mitochondrial-targeted restriction endonuclease to induce *mt:CoI^ts^*, a point mutation, specifically in the retina of *Drosophila*, leaving mitochondrial function in other tissues unaffected (30). At 29°C, these heterozygous flies exhibited severe optic nerve degeneration, with mitochondrial calcium dysregulation playing a key role in the degeneration process. While this study provided valuable insights into mitochondrial mutation mechanisms, it focused primarily on the novel genetic methodology and did not explore in detail the impact of mitochondrial dysfunction on visual signal transduction.

### The progressive impact of mitochondrial dysfunction on vision

Our study reveals that mitochondrial dysfunction significantly impacts the fly’s response to light stimuli, as measured by electroretinogram (ERG) recordings. At 18°C, *mt:CoI^ts^* flies show normal mitochondrial function and ERG responses. However, when reared at 29°C, we found a progressive decline in ERG amplitudes and an increased delay in photoresponse termination in *mt: CoI^ts^* mutants, which suggests that mitochondrial dysfunction impairs both the light activation and deactivation phases of visual signal transduction. This is consistent with previous reports linking mitochondrial defects to alterations in the kinetics of photoreceptor responses (41). In particular, we noted a pronounced reduction in the off-transient component of the ERG, reflecting impaired postsynaptic signaling within the retina. These findings suggest that the impact of mitochondrial dysfunction on visual signal transduction is a progressive process that exhibits a threshold effect. Prior to reaching this threshold, visual function remains largely unaffected; once mitochondrial dysfunction surpasses a certain level, ERG abnormalities become apparent, including diminished amplitude and delayed deactivation.

### Progressive mitochondrial dysfunction leads to retinal degeneration

Mitochondrial dysfunction also parallels gene mutations in causing retinal degeneration. Numerous studies have shown that mutations affecting visual signal transduction, such as those in *ninaE*, *Gq*, *MPPE*, *noprA*, and *rdgA*, ultimately result in retinal degeneration (38, 39, 42–44). The onset of retinal degeneration varies with the mutation: some mutations (e.g., *noprA*, *rdgA*) cause rapid degeneration within 24 hours, while others take up to 20 days to manifest (43, 44). In contrast, retinal degeneration induced by mitochondrial dysfunction occurs more gradually (38, 39). Electron microscopy revealed that when mitochondrial function in *mt:CoI^ts^* flies is reduced to approximately 20% of wild-type levels, retinal degeneration becomes evident, although it remains milder compared to the degeneration caused by genetic mutations. These observations suggest that retinal degeneration due to mitochondrial dysfunction is more insidious and may be easily overlooked, especially in its early stages.

Mitochondrial dysfunction represents one of the key mechanisms underlying neurodegeneration in retinal diseases. Although different genetic mutations can lead to mitochondrial dysfunction, the phenotypic outcomes vary. Previous studies have demonstrated that mutations in numerous genes can impair mitochondrial function, leading to an increase in ROS content, which in turn induces retinal degeneration (41, 45). Notably, In the *mt:CoI^ts^* mutant flies, elevated levels of reactive oxygen species (ROS) are not observed. Instead, retinal degeneration is attributed to the formation of a stable complex between Arr2 and Rh1, which hinders their dissociation and consequently initiates the process of retinal degeneration. This mechanism elucidates an alternative pathway to ROS-mediated degeneration, highlighting the complexity of genetic interactions in the context of retinal health.

Our study underscores the profound impact of progressive mitochondrial dysfunction on visual transduction, culminating in decreased light sensitivity, reduced amplitude, and ultimately retinal degeneration. Furthermore, we highlight that mitochondrial dysfunction exhibits a threshold effect, with phenotypic changes occurring only after a certain degree of dysfunction is reached. This progressive and less detectable nature of mitochondrial dysfunction makes its early detection particularly challenging compared to the more rapid onset of defects associated with genetic mutations. Therefore, understanding the subtle and gradual effects of mitochondrial dysfunction on visual health requires more comprehensive research approaches and greater attention to the interplay between mitochondrial health and physiological function.

In conclusion, our study highlights the critical role of mitochondrial function in maintaining visual signal transduction and retinal integrity. Our findings provide compelling evidence that mitochondrial dysfunction leads to a cascade of events that disrupt visual processing, decrease light sensitivity, and promote retinal degeneration. Future research should focus on elucidating the precise molecular pathways linking mitochondrial dysfunction to retinal degeneration, as well as exploring potential therapeutic strategies to mitigate the effects of mitochondrial defects on retinal health.

## Data Availability

The original contributions presented in the study are included in the article/supplementary material. Further inquiries can be directed to the corresponding authors.
